# Krankheitsmodifizierende Therapieansätze bei Alzheimer-Krankheit

**DOI:** 10.1007/s00115-021-01222-w

**Published:** 2021-11-04

**Authors:** Lutz Frölich, Lucrezia Hausner

**Affiliations:** Zentralinstitut für Seelische Gesundheit Mannheim, Abteilung Gerontopsychiatrie, Universität Heidelberg, Quadrat J5, 68159 Mannheim, Deutschland

**Keywords:** Neuroprotektion, Behandlung, Amyloid, Tau, Antikörper, Neuroprotection, Treatment, Amyloid, Tau, Antibody

## Abstract

Die Alzheimer-Krankheit ist eine der großen Volkskrankheiten mit bisher allein symptomatischen Therapieoptionen. Neue Erkenntnisse zu einem Krankheitskontinuum mit einer sehr langen präklinischen und frühsymptomatischen Krankheitsphase sowie molekulare Therapiestrategien, die auf den Erkenntnissen zur molekularen Neurobiologie der Erkrankung fußen, eröffnen eine Vielzahl neuer therapeutischer Strategien. Im Jahr 2021 ist erstmals ein Antiamyloidantikörper in den USA als krankheitsmodifizierende Therapie bei Alzheimer-Krankheit zugelassen worden, was einen ersten, sehr kontrovers diskutierten Schritt hin zu einer molekularen, ursachenorientierten Therapie darstellt. Die Übersicht stellt die am weitesten entwickelten molekularen Therapiestrategien sowie die Auswirkungen der zugelassenen Antikörpertherapie für die Praxis vor. Die Besonderheiten einer Langzeittherapie mit i.v. Infusionen in einer besonders vulnerablen Patientenpopulation und einem speziellen Nebenwirkungsprofil wird für die Implementierung in die Praxis große Herausforderungen mit sich bringen und ein hohes Maß an Kooperation erfordern. Die Zukunft der Alzheimer-Therapie mit einem multimodalen Therapieansatz mit verschiedenen Substanzen wird diesen Trend wahrscheinlich noch verstärken.

## Abstract (en)

Alzheimer’s disease is one the major common diseases but so far only with symptomatic treatment options. New insights define the disease as a slowly progressive continuum with very long preclinical and early symptomatic phases. Innovative molecular treatment strategies are based on an improved understanding of the molecular neurobiology of the disease, opening up a variety of therapeutic targets. For the first time, an anti-amyloid antibody has been approved in the USA in 2021 as a disease-modifying treatment for Alzheimer’s disease, representing a first highly controversial step towards a molecular, cause-oriented treatment. This review presents the most advanced molecular treatment strategies and discusses the implications of the approved antibody treatment for the clinical practice. The special features of this long-term treatment with i.v. infusions in a particularly vulnerable population and a special side effect profile will impose significant challenges for implementation in the practice and will require a high degree of cooperation within the healthcare system. The future of Alzheimer’s treatment with a multimodal therapeutic approach with different classes of drugs will probably reinforce these trends.

## Hintergrund

Die Alzheimer-Krankheit (AK) ist eine der großen Volkskrankheiten, somit häufig, chronisch progredient, schwerwiegend und kostenträchtig. In Deutschland sind derzeit rund 700.000 Menschen an einer Alzheimer-Demenz (AD) erkrankt, damit ist sie die häufigste Form der Demenzerkrankungen. Jedes Jahr erkranken etwa 200.000 Menschen neu [14]. Der Alzheimer-Demenz geht eine 15- bis 20-jährige präklinische Phase voraus sowie eine 3‑ bis 6‑jährige Prodromalperiode vor der Demenzmanifestation [30].

Bisherige pharmakologische Therapiemöglichkeiten bei der AK sind rein symptomatischer Natur und die verfügbaren Substanzen basieren auf Wirkmechanismen der Neurotransmittermodulation [27]. Die derzeitigen innovativen Therapiestudien bei AK gehen von Konzepten der Krankheitsmodifikation aus, d. h. sie postulieren eine therapeutische Beeinflussung grundlegender Mechanismen der Ätiopathogenese der AK. Es wurden über spezifische pathogenetische Mechanismen, welche über experimentelle, molekularpathologische Biomarkerveränderungen an Patienten identifiziert wurden, pharmakologische Ziele abgeleitet und Substanzen entwickelt. Diese Wirkmechanismen unterscheiden sich von älteren Konzepten der Neuroprotektion bei chronisch neurodegenerativen Erkrankungen, die sich vorwiegend auf Befunde aus experimentellen Forschungsparadigmen stützen und weniger über Biomarkerbefunde an Patienten belegt sind. Dies erhöht die Wahrscheinlichkeit, dass eine Translation experimenteller Befunde in klinische Studienergebnisse gelingt.

Die Pathobiologie der AK wird wesentlich als komplexe zerebrale Proteinopathie zweier Proteinsysteme verstanden: Nach der Amyloid-Kaskaden-Hypothese [28] und ihren Erweiterungen [6] stellt die Amyloidproteinopathie das erste zentrale Ereignis der Pathophysiologie dar, das mit der Tau-Proteinopathie interagiert [3, 4] und dann eine Vielzahl weiterer molekularer Kaskaden anstößt, die zu synaptischer Dysfunktion und Neurodegeneration führen [25]. Aus diesen Erkenntnissen wurden entsprechend zwei spezifische molekular definierte Therapiestrategien abgeleitet:

1. die Entstehung pathologischer Amyloidaggregate und amyloider Plaques zu reduzieren und
2. die Entstehung hyperphosphorylierten Tau-Proteins und dessen Aggregation zu neurofibrillären Bündeln zu reduzieren.

Die sporadische AK ist zusätzlich eine sehr stark altersassoziierte Erkrankung, und das Gehirn ist als postmitotisches Organ besonders empfindlich gegenüber Alterungseffekten. Deswegen werden spezifische molekulare Mechanismen der Hirnalterung, wie genomische Instabilität, Mitophagie, zelluläre Seneszenz, Proteinaggregation, mitochondriale Dysfunktion und Neuroinflammation auch als therapeutische Ziele bei AK erforscht. [8, 15].

Die Auswahl der klinischen Studien für die vorliegende Übersicht erfolgte nach einem systematischen Vorgehen und war nicht primär am Wirkmechanismus der Substanzen orientiert: Ausgehend von einer systematischen Analyse der öffentlich zugänglichen klinischen Therapiestudien (Datenbank: FDA/US National Library of Medicine of the National Institutes of Health Clinicaltrials.gov; [8]) wurden die derzeit laufenden oder abgeschlossenen klinischen Studien identifiziert. Daraus wurden wegen der Praxisrelevanz die am weitesten fortgeschrittenen Studien (laufende oder abgeschlossene Phase-3-Studien) ausgewählt (Tab. 1) und die Substanzkandidaten vorgestellt. Dies impliziert, dass hoch innovative, aber (noch) nicht klinisch relevante Ansätze wie gentherapeutische oder stammzellbezogene Ansätze hier nicht vorgestellt werden, ebenso wie wegen inadäquater klinischer Studienlage manche Ansätze mit natürlich vorkommenden Substanzen. Zusätzlich werden die absehbaren Auswirkungen der ersten zugelassenen Amyloidantikörpertherapie für die klinische Versorgung bei AK diskutiert.

**Table 1:** 

Wirkstoff	Therapeutisches Ziel (CADRO-Klassifikation)	Wirkmechanismus	Status der Studie (ID-Nr. des Clinical Trials.gov Register)	Sponsor
Aducanumab	Amyloid	Monoklonaler Antikörper gegen Aβ-Plaques und -Oligomere	Rekrutierung (nur nach Einladung) (NCT04241068)	Biogen
AGB101 (niedrig dosiertes Levetiracetam)	Synaptische Plastizität/Neuroprotektion	SV2A-Modulator; Reduzierung der Aβ-induzierten neuronalen Hyperaktivität	Rekrutierung (NCT03486938)	AgeneBio, NIA
Atuzaginstat (COR388)	Inflammation/Infektion	Bakterieller Proteaseinhibitor (gegen Gingipain, produziert durch *P. gingivalis*) soll die Neuroinflammation reduzieren	Aktiv, Rekrutierung beendet (NCT03823404)	Cortexyme
Blarcamesine (ANAVEX2-73)	Synaptische Plastizität/Neuroprotektion	Sigma-1-Rezeptor-Agonist, M2-Autorezeptor-Antagonist, gegen oxidativen Stress, Proteinfehlfaltung, mitochondriale Dysfunktion und Inflammation	Rekrutierung (NCT03790709)	Anavex Life Science
Gantenerumab	Amyloid	Monoklonaler Antikörper gegen Aβ-Plaques und -Oligomere	Aktiv, Rekrutierung beendet (NCT02051608) (NCT03444870) (NCT03443973) (NCT04339413)	Roche
Gantenerumab und Solanezumab	Amyloid	Monoklonaler Antikörper gegen Aβ-Plaques und -Oligomere (Gantenerumab); monoklonaler Antikörper gegen Aβ-Monomere (Solanezumab)	Rekrutierung (NCT01760005)	Washington University, Eli Lilly, Roche, NIA, AA
GV-971	Inflammation/Darm-Gehirn-Achse	Aus Algen gewonnene säurehaltige Oligosaccharide; antientzündliche Veränderung des Mikrobioms	Rekrutierung (NCT04520412)	Shanghai Green Valley
Lecanemab (BAN2401)	Amyloid	Monoklonaler Antikörper gegen Aβ-Protofibrillen	Rekrutierung (NCT03887455)	Eisai, Biogen
Metformin	Stoffwechsel und Bioenergetik	Insulin-Sensitisierer, Verbesserung des ZNS-Glukosestoffwechsels	Noch nicht rekrutierend (NCT04098666)	Columbia University, NIA
NE3107	Inflammation	MAPK-1/3-Inhibitor; reduziert proinflammatorische NFκB-Aktivierung	Noch nicht rekrutierend (NCT04669028)	Neurmedix
Tricaprilin	Stoffwechsel und Bioenergetik	Capryl-triglycerid; induziert Ketose, verbessert mitochondriale Dysfunktion	Noch nicht rekrutierend (NCT04187547)	Cerecin
Troriluzol (BHV4157)	Synaptische Plastizität/Neuroprotektion	Glutamatmodulator; Prodrug des Riluzol, Verbesserung neuronaler Funktion	Aktiv, Rekrutierung beendet (NCT03605667)	Biohaven Pharm., ADCS
TRx0237	Tau	Tau-Protein-Aggregation Hemmstoff	Aktiv, Rekrutierung beendet (NCT03446001)	TauRx Therapeutics

*CADRO* Common Alzheimer’s Disease Research Ontology, *ZNS* Zentralnerensystem

## Grundlegende ätiopathogenetische Mechanismen mit Einfluss auf die Proteinopathie bei AK

Die zentrale Rolle der Amyloidpathologie in der Pathogenese der AK ist durch viele experimentelle, pathologische, genetische und Biomarkerstudien sowohl bei der familiären (früh einsetzenden) als auch bei der sporadischen (später einsetzenden) AK bestätigt. Die Amyloid-Kaskaden-Hypothese integriert diese Befunde in ein komplexes Modell der Ätiopathogenese [28] und ist derzeit der wichtigste Ausgangspunkt zur Identifizierung pharmakologischer Ziele für krankheitsmodifizierende Therapien [12]. Im Zentrum der Hypothese steht eine Störung des Metabolismus des Amyloidvorläuferproteins (Amyloid-Precursor-Protein, APP). Dies führt zur Generierung mehrerer Isoformen des β‑Amyloid-Peptids unterschiedlicher Länge, die zu zunächst löslichen toxischen Polymeren autoaggregieren. Die Amyloidoligomere sind das derzeit wichtigste pharmakologische Ziel. Die schließlich entstehenden amyloiden Plaques werden extrazellulär abgelagert und sind Kristallisationspunkt für vielfältige neuroinflammatorische Reaktionen.

„Tau-Seeds“ überschreiten zelluläre Barrieren und „infizieren“ gesunde Zellen

Ausgehend von diesem Modell werden vielfältige Rückschläge bei der Therapieentwicklung v. a. auf Schwächen in der Konzeption, Durchführung und Auswertung der klinischen Studien zurückgeführt. Die Rückschläge führten aber auch zu einer intensiven Diskussion um die Validität des ätiopathogenetischen Modells [13]. Für dieses Modell spricht auch, dass die sog. isländische Mutation, eine genetische APP-Variante, die zu einem Rückgang der Amyloidproduktion um etwa 40 % führt, mit einem geringeren Alzheimer-Risiko assoziiert ist [19]. Auf der Basis der Amyloid-Kaskaden-Hypothese lassen sich die Ergebnisse vieler klinischer Verlaufsstudien mit Biomarkern in ein diagnostisches Rahmenkonzept mit Relevanz für klinische Studien integrieren, wonach das Auftreten zerebraler Amyloidablagerungen der Tau-Pathologie vorangeht und somit als notwendige Bedingung für die Progression der AK gilt [17]. Amyloideplaques sind aber möglicherweise nur eine zelluläre Reaktion, durch die lösliche, toxische Amyloidoligomere sequestriert und neutralisiert werden [6], weshalb Oligomere derzeit für das effektivere therapeutische Ziel gehalten werden.

In den derzeitigen Modellvorstellungen geht man von komplexen Interaktionen zwischen Amyloid- und Tau-Pathologie aus, die den Krankheitsprozess der AK aufrechterhalten und vorantreiben [24]. Das mikrotubulusassoziierte Protein Tau (MAP-Tau) ist ein intrazelluläres, komplex reguliertes und translational vielfältig modifiziertes Protein, das axonale Transportmechanismen stabilisiert. Hyperphosphorylierte Formen von Tau sind instabil und aggregieren. Intraneuronal entstehen zunächst Aggregate pathologisch veränderten Tau-Proteins („seeds“), diese können zelluläre Barrieren überschreiten und über einen prionenartigen Mechanismus gesunde Zellen „infizieren“. In der Folge breitet sich die Tau-Pathologie entlang neuronaler Bahnen im Gehirn aus, wobei die von den betroffenen Neuronen produzierten „Tau-Seeds“ in gesunde, aber anfällige Zellen eindringen, wo sie physiologisches Tau über eine Konformationsänderung sequestrieren und umwandeln. Neuronale Schäden entstehen durch eine Kombination aus Verlust physiologischer Funktionen und Zunahme toxischer Funktionen des veränderten Tau-Proteins [3]. Es ist lange bekannt, dass nicht amyloide Plaques, sondern das Ausmaß der neurofibrillärer Degeneration mit dem Grad der kognitiven Störung korreliert und prädiktiv für den weiteren klinischen Verlauf ist [4]. Hieraus pharmakologische Ziele zu definieren, ist wegen der Komplexität und der intrazellulären Lokalisation des Tau-Proteins sowie der noch lückenhaften Kenntnisse zu den Mechanismen der pathologischen Tau-Ablagerung deutlich schwieriger als beim Amyloid [10].

**Figure 1:**
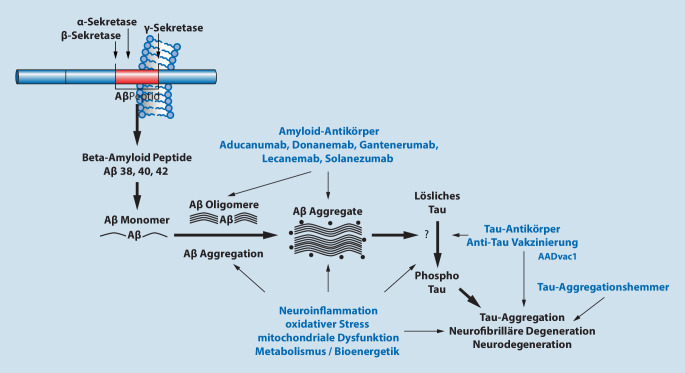

Die zeitliche Dynamik der proteinbezogenen ätiopathogenetischen Veränderungen wird als entscheidend für die klinische Manifestation der AK angesehen. Denn Erkenntnisse zu krankheitsassoziierten Genen sowie experimentelle Befunde zur Rolle mikrogliavermittelter Neuroinflammation belegen, dass über die Proteinaggregate angestoßene Veränderungen des Immunsystems ein wichtiger pathogenetischer Faktor als Motor der Neurodegeneration sind und entscheidend die Symptomatik und den Verlauf der Erkrankung bestimmen [5]. Eine schematische Illustration der ätiopathogenetischen Hypothesen der Alzheimer-Krankheit und daraus resultierender pharmakologischer Angriffspunkte ist in Abb. 1 dargestellt.

## Wirkstoffe in fortgeschrittener klinischer Entwicklung (Phase 3)

Die Arzneimittelentwicklung bei AK fokussiert v. a. auf Wirkmechanismen, die nach der CADRO(Common Alzheimer’s Disease Research Ontology)-Klassifikation als krankheitsmodifizierend bewertet werden [16]. Diese Wirkstoffe befinden sich zumeist in Studien zur Sekundärprävention bei Patienten mit präklinischer oder prodromaler AK sowie leichter AD [25]. Verschiedene therapeutische Ansätze wurden wegen Negativergebnissen wieder aufgegeben, z. B. β‑ und γ‑Sekretase-Inhibitoren [12]. Viele hoch innovative therapeutische Ansätze befinden sich erst in frühen klinischen Stadien der Medikamentenentwicklung (Phase 1–2). Viele dieser Entwicklungen werden aufgrund negativer Studienlage im Verlauf der Phase 2 aus unterschiedlichen Gründen wieder eingestellt, sodass sich Fragen zu klinisch relevanten Ergebnissen (noch) nicht stellen. Deswegen wird diese Studienlage hier nicht diskutiert.

Im US-amerikanischen Studienregister (FDA/US National Library of Medicine of the National Institutes of Health Clinicaltrials.gov) werden 2021 14 Wirkstoffe in Phase-3-Studien an Patienten mit einer symptomatischen AK (im Stadium der leichten kognitiven Störung oder leichten-mäßigen Demenz) aufgeführt (Tab. 1). Darunter befinden sich 4 Biologika und 10 orale Wirkstoffe/kleine Moleküle. Alle 4 Biologika haben Amyloid als primären oder hauptsächlichen Angriffspunkt. Andere pharmakologische Targets nach der CADRO-Klassifikation sind Tau-Pathologie (ein Wirkstoff), Entzündung/Infektion (3 Wirkstoffe, davon einer mit Wirkmechanismus über die Darm-Hirn-Achse), Stoffwechsel und Bioenergetik (3 Wirkstoffe) und synaptische Plastizität/Neuroprotektion (3 Wirkstoffe). Vier der Wirkstoffe sind bereits für andere Indikationen zugelassen („repurposed drugs“; [8]).

Neben den umfangreichen Studien an symptomatischen AK-Patienten laufen mehrere analoge Phase-3-Präventionsstudien an kognitiv unbeeinträchtigten Risikopopulationen (präklinische AK). Eine der Phase-3-Studien rekrutiert wegen der speziellen Probandenpopulation (Mutationsträger) sowohl präklinische Patienten als auch Patienten mit „mild cognitive impairment“/leichter AD (DIAN-TU-Studie; [8]).

Lecanemab zeigte klinische Effekte in kognitiven Tests

Die Antiamyloidansätze in Phase 3 umfassen ausschließlich monoklonale Antikörper. Die Studien mit *Aducanumab* sind abgeschlossen (siehe unten). *Solanezumab* wird nach negativen Phase-3-Studien an symptomatischen Alzheimer-Patienten [13] nur noch für eine präventive Indikation untersucht. *Gantenerumab* wird nach ersten negativen Phase-3-Studien in höherer Dosierung und in Studien längerer Dauer weiterentwickelt [23]. *Lecanemab* zeigte in einer Phase-2-Studie nach 18 Monaten eine signifikante Reduktion der Amyloidlast im Gehirn und klinische Effekte in kognitiven Tests und der globalen klinischen Einschätzung. Liquorbiomarker sprachen zusätzlich für einen Behandlungseffekt auf Neurodegeneration [29]. Dieser Antikörper wird derzeit in einer Phase-3-Studie konfirmatorisch auf Wirksamkeit geprüft. In einer Phase-2-Studie zeigte *Donanemab* bei Patienten im Frühstadium der AK nach 18 Monaten positive Effekte auf Kognition und Alltagskompetenz gegenüber Placebo [19]. Eine Phase-3-Studie ist gerade gestartet.

Alle bisherigen Tau-Antiköper-Studien gingen negativ aus [18]. Viele kleine Moleküle zur Verringerung der Tau-Pathologie sind in der Entwicklung. Darunter sind Modulatoren der posttranslationalen Modifikation, Aggregationshemmer und Abbaupromotoren, meist noch im präklinischen Stadium. Eine Phase-3-Studie zu dem Aggregationshemmer *TRx0237 *läuft derzeit noch [33]. Eine aktive Vakzine (AADvac1) wurde in einer klinischen Phase-2-Studie mit Erfolg getestet [22]. Eine Phase-3-Studie mit der Substanz ist in Vorbereitung.

Oligomannat ist eine in China zugelassene Substanz, die in einer Phase-3-Studie eine Verbesserung der Kognition gezeigt hatte. Als Wirkmechanismus im Darmmikrobiom wird angenommen, dass die Substanz die Zusammensetzung der Darmflora normalisiert und dann über periphere Entzündungsmediatoren die Neuroinflammation reduziert [32].

## Zulassung des ersten Amyloidantikörpers als krankheitsmodifizierende Therapie in den USA

Im Juni 2021 hat die US-Arzneimittelbehörde FDA Aducanumab für die Behandlung der leichten AK zugelassen (Dosierung: 10 mg/kg Körpergewicht, monatliche i.v. Infusion). Der Hersteller wurde verpflichtet, nach der Zulassung eine zusätzliche konfirmatorische klinische Studie zum klinischen Nutzen von Aducanumab vorzulegen. Sollte der Nutzen nicht belegt werden können, kann die FDA die Zulassung widerrufen.

Diese Zulassung markiert einen Wendepunkt in der psychiatrischen Pharmakotherapie, da erstmals eine Substanz gegen die grundlegende Pathophysiologie einer Erkrankung aufgrund relevanter Effekte auf Biomarkerebene zugelassen wurde, jedoch nicht aufgrund klinischer Belege. Diese sollen erst durch die Ergebnisse einer bis 2030 vorzulegenden zusätzlichen Studie bestätigt werden.

Die Zulassung von Aducanumab erfolgte aufgrund relevanter Effekte auf Biomarkerebene

Die bisherigen klinischen Studiendaten waren uneindeutig und mit operationalen Schwächen behaftet. Eine Phase-1-Studie zur Untersuchung der Sicherheit von Aducanumab hatte eine signifikante Reduktion der Amyloidlast im Gehirn gezeigt und die klinischen Daten legten nahe, dass der kognitive Abbau dosisabhängig verlangsamt wurde. Wegen der positiven Ergebnisse wurden die sonst üblichen Phase-2-Studien übersprungen und sofort zwei Phase-3-Studien mit jeweils etwa 1640 Patienten begonnen. Diese Studien wurden Anfang März 2019 nach einer negativen Zwischenanalyse gestoppt. Infolgedessen konnten 37 % der Teilnehmer die 78-wöchige Studiendauer nicht abschließen. Im Oktober 2019 wurde bekannt gegeben, dass nach einer neuen Zwischenauswertung doch Beweise für Wirksamkeit vorliegen. Diese Schlussfolgerung bezog Daten von weiteren 318 Teilnehmern ein, die vor dem Abbruch der Studien, aber nach dem Stichtag der Zwischenauswertung erhoben wurden. In einer der beiden Studien führte die höchste Dosis (10 mg/kg Körpergewicht) zu einer signifikanten Verlangsamung des kognitiven Abbaus um 22 % im Vergleich zu Placebo. Eine niedrigere Dosis in dieser Studie und beide Dosierungen in der zweiten Studie zeigten keine signifikante Überlegenheit gegenüber Placebo. Nur eine Post-hoc-Analyse der Teilnehmer der Untergruppe mit hoher Dosis und längster Behandlungsdauer in der insgesamt negativen zweiten Studie ergab Hinweise auf Wirksamkeit. Die signifikante Reduktion der Amyloidlast im Gehirn wurde in beiden Phase-3-Studien bestätigt und war die Grundlage der Zulassung. Die klinische Relevanz dieser Ergebnisse wurde sehr kontrovers diskutiert.

Die häufigste Nebenwirkung von Aducanumab waren amyloidbezogene Bildgebungsanomalien (ARIA) in der Magnetresonanztomographie (MRT) bei 35,2 % der Patienten in der Hochdosisgruppe im Vergleich zu 2,7 % in der Placebogruppe. Das sind vorübergehende, meist asymptomatische Schwellungen oder fokale zerebrale Mikroblutungen (74 % asymptomatisch). Typische Symptome von ARIA waren Verwirrtheit, Delir, Schwindel oder Sehstörungen (in 67,7 % leicht, in 4 % schwer ausgeprägt). Weitere Nebenwirkungen waren Stürze und Diarrhö und sehr selten allergische Reaktionen [8].

## Klinische Voraussetzungen der therapeutischen Anwendung in der Praxis

Die Anwendung von Aducanumab in der Praxis wird ähnliche Herausforderungen mit sich bringen wie andere i.v. Infusionstherapien, mit den Besonderheiten einer aufwendigen Eingangsdiagnostik und einem langfristigen Monitoring auf Nebenwirkungen mittels MRT bei einer besonders vulnerablen Population von Menschen im meist hohen Alter. Für das praktische Vorgehen wurden kürzlich Expertenempfehlungen vorgelegt [7], die entlang der Eingangskriterien für die Zulassungsstudien formuliert wurden (Tab. 2). Hierzu gehören der Nachweis leichter kognitiver Defizite sowie der positive Nachweis der zerebralen Amyloidpathologie. Aducanumab soll über einen Zeitraum von 6 Monaten in mehreren Stufen auf eine Dosis von 10 mg/kg titriert werden, unter sorgfältigem MRT-Monitoring für ARIA vor Beginn und mindestens 4‑mal im ersten Behandlungsjahr. Bei symptomatischen oder mittelgroßen ARIA ohne Symptome soll die Behandlung nach einem bestimmten Algorithmus unterbrochen, kontrolliert und wiederaufgenommen bzw. beendet werden. Eine ausführliche regelmäßige Beratung der Patienten und ihrer Bezugspersonen und deren Beteiligung an den Therapieentscheidungen werden gefordert. Dies setzt entsprechende Kompetenzen auf allen Seiten voraus.

Derzeit ist vorgesehen, die Behandlung bis in das mittlere Demenzstadium fortzuführen. Sobald eine signifikante Absenkung der Amyloidlast erreicht ist, könnte die Häufigkeit der Infusionen verringert werden. Das impliziert möglicherweise wiederholte Amyloid-Positronenemissionstomographien oder Liquorpunktionen. Eine solche dauerhafte Amyloidabsenkung ist bisher für Donanemab [21], aber nicht für Aducanumab belegt.

**Table 2:** 

Patientenkriterien	Voraussetzungen zur Anwendung in der klinischen Praxis
Alter	Keine Altersbeschränkung
Diagnose	Klinische Diagnosekriterien entsprechend den NIA-AA-Kriterien [1, 20]: Leichte kognitive Störung bei Alzheimer-Krankheit oder leichte Alzheimer-Demenz
Schweregrad der kognitiven Störung (Skalenwerte)	Psychometrische Kurztests: MMSE 21–30 Punkte oder analog MoCA 17–30 Punkte
Amyloid Status	Positiver Amyloid-PET-Befund (visuelle Auswertung) oder Liquor Alzheimer-Biomarker vereinbar mit Alzheimer-Krankheit
Genetische Testung auf APO-E-Polymorphismus	Aufklärung von Patient und Bezugsperson über Nutzen und Risiken der APO-E-Genotypisierung, spezifische Aufklärung über differenzielle ARIA-Risiken, partizipative Entscheidung nach Patientenpräferenz
Neurologischer Status	Ausschluss von Non-Alzheimer-Demenzerkrankungen
Psychiatrischer Status	Psychiatrisch stabil und kooperationsfähig, nach klinischer Beurteilung keine Risiken bei der Mitwirkung am therapeutischen Regime
Kardiovaskuläre Vorerkrankungen	Stabiler kardiovaskulärer Status, nach klinischer Beurteilung keine Risiken bei Durchführung des therapeutischen Regimes
Medizinische Vorgeschichte	Stabiler allgemeinmedizinischer Status, nach klinischer Beurteilung keine Risiken bei Durchführung des therapeutischen Regimes
Gerinnungsstatus	Keine Antikoagulation
Reproduktiver Status	Ausschluss von Schwangeren oder stillenden Frauen, Frauen im gebärfähigen Alter nur mit Verhütung
Antidementive Medikation	Acetylcholinesteraseinhibitoren, Memantin erlaubt
MRT-Befund	Ausschluss bei Nachweis einer akuten oder subakuten Blutung oder Makrohämorrhagie, > 4 Mikroblutungen, kortikaler Infarkt (> 1,5 cm), 1 lakunärer Infarkt (> 1,5 cm), > 1 Bereich mit superfizieller Siderose oder diffuse Schädigung der weißen Substanz
Pflegerische Unterstützung	Selbständig oder mit pflegerischen Hilfen
Einwilligung	Einwilligungsfähig für Art und Voraussetzungen der Therapie (monatliche Infusionen ohne zeitliche Befristung) sowie für Kriterien des Therapieerfolg (Verlangsamung des kognitiven Abbaus)

*APO‑E *Apolipoprotein E, *ARIA *amyloidbezogene Bildgebungsanomalien, *MMSE *Mini-Mental State Examination, *MoCA *Montreal Cognitive Assessment,* MRT *Magnetresonanztomographie, *NIA-AA *National Institute on Aging and Alzheimer‘s Association, *PET* Positronenemissionstomographie

## Perspektiven und Konsequenzen

Der beschleunigte Zulassungsweg der FDA ist für Medikamente gegen schwere Krankheiten vorgesehen, von denen ein bedeutender Vorteil gegenüber den verfügbaren Therapien erwartet wird, auch wenn eine Restunsicherheit über den endgültigen klinischen Nutzen des Medikaments besteht. Dafür muss ein substanzieller Beweis für die Wirksamkeit des Medikaments auf einem „Surrogat-Endpunkt“ vorliegen – in der Regel ein Biomarker, der die zugrunde liegende Pathologie widerspiegelt. Die Wirkung auf den Surrogat-Endpunkt muss plausibel einem entsprechenden klinischen Nutzen entsprechen. Ob dies für die zerebrale Amyloidablagerung bei Alzheimer gilt, wird kontrovers diskutiert [2, 9, 11, 26]. Aducanumab ist aktuell nur für die USA zugelassen. Wie die europäische Zulassungsbehörde EMA entscheiden wird, ist unsicher, weil in Europa ein analoger Zulassungsweg nicht existiert. Die Zulassung von Aducanumab in Europa ist jedoch beantragt. Auch die drei weiteren o. g. Amyloidantikörper streben eine Zulassung in den USA nach demselben Zulassungsweg wie Aducanumab an.

In der klinischen Alzheimer-Forschung sind große Fortschritte zu verzeichnen wie neue Krankheitskonzepte, Früherkennung und Demenzprädiktion, die Biomarkerentwicklung inklusive blutbasierter Biomarker [31]. Es ist zu erwarten, dass weitere krankheitsmodifizierende Therapien mit anderen Wirkmechanismen zur Zulassung kommen. Die Zukunft der Alzheimer-Behandlung liegt wahrscheinlich in einem multimodalen Therapieansatz mit verschiedenen Substanzen, ähnlich wie bei Chemotherapien. Noch ist nicht klar, welche Substanzen in welcher Reihenfolge sinnvoll kombiniert und wie geeignete Patientengruppen definiert werden können. Diese Trends werden uns neue Arbeitsweisen mit einem hohen Maß an Kooperation abverlangen.

## Fazit für die Praxis

- Die komplexe Proteinopathie (Amyloido- und Tauopathie) bei Alzheimer-Krankheit (AK) ist derzeit der wichtigste Ausgangpunkt für innovative Therapieansätze. Darüber hinaus werden Ansätze gegen Neuroinflammation und Mechanismen der Hirnalterung therapeutisch beforscht.
- Ein erster monoklonaler Amyloidantikörper ist zur Behandlung der prodromalen AK und leichten Demenz bei Alzheimer-Krankheit in den USA zugelassen worden. Dessen klinische Wirksamkeit muss sich in der Praxis noch beweisen.
- Wegen der vulnerablen Patientenpopulation und der hohen Patientenzahl ergeben sich zahlreiche praktische Herausforderungen für diese neuen Therapien, ethische Fragen sowie notwendige Regelungen in Bezug auf Allokation und Zugänglichkeit.
- Es ist unerlässlich, alternative Wege der Pathogenese (u. a. oxidativer Stress, Neuroinflammation, neuronale Plastizität, vaskuläre Faktoren) als mögliche therapeutische Targets zu erforschen.
- Um diese Therapien erfolgreich zu implementieren, bedarf es neuer Wege der Kooperation zwischen niedergelassenen Nervenärzten und Expertenzentren.

## References

[1] Albert MS (2011). The diagnosis of mild cognitive impairment due to Alzheimer’s disease: recommendations from the National Institute on Aging-Alzheimer’s Association workgroups on diagnostic guidelines for Alzheimer’s disease. Alzheimers Dement.

[2] Alexander GC, Emerson S, Kesselheim AS (2021). Evaluation of aducanumab for alzheimer disease: scientific evidence and regulatory review involving efficacy, safety, and futility. JAMA.

[3] Arendt T, Stieler JT, Holzer M (2016). Tau and tauopathies. Brain Res Bull.

[4] Braak H, Feldengut S, Del Tredici K (2013). Pathogenese und Prävention des M. Alzheimer: Wann und auf welche Weise beginnt der pathologische Prozess?. Nervenarzt.

[5] Castro-Gomez S, Binder J, Heneka MT (2019). Neuroinflammation als Motor der Alzheimer-Erkrankung. Nervenarzt.

[6] Clein NE, Bicca MA, Viola KL, Klein WL (2018). The amyloid-oligomer hypothesis: beginning of the third decade. J Alzheimer Dis.

[7] Cummings J, Aisen P, Apostolova LG (2021). Aducanumab: appropriate use recommendations. J Prev Alzheimers Dis.

[8] Cummings J, Lee G, Zhong K (2021). Alzheimer’s disease drug development pipeline: 2021. Alzheimers Dement.

[9] Dunn B, Stein P, Cavazzoni P (2021). Approval of aducanumab for alzheimer disease—the FDA’s perspective. JAMA Intern Med..

[10] Fließbach K, McCormick C, Kaulen B, Schneider A (2019). Anti-Tau-Therapien – was können wir erwarten?. Nervenarzt.

[11] Gyawali B, Ross JS, Kesselheim AS (2021). Fulfilling the mandate of the US food and drug administration’s accelerated approval pathway: the need for reforms. JAMA Intern Med.

[12] Haass C, Levin J (2019). Hat die Alzheimer-Forschung versagt? Das Scheitern amyloid-basierter klinischer Studien. Nervenarzt.

[13] Honig LS, Vellas B, Woodward M (2018). Trial of solanezumab for mild dementia due to alzheimer’s disease. N Engl J Med.

[14] https://www.deutsche-alzheimer.de/fileadmin/alz/pdf/factsheets/infoblatt1_haeufigkeit_demenzerkrankungen_dalzg.pdf. Zugegriffen: 29. Juli 2021

[15] Hou Y, Dan X, Babbar M, Wei Y (2019). Ageing as a risk factor for neurodegenerative disease. Nat Rev Neurol.

[16] International Alzheimer’s and Related Dementia Research Portfolio https://iadrp.nia.nih.gov/about/cadro. Zugegriffen: 29. Juli 2021

[17] Jack CR, Bennett DA, Blennow K (2018). NIA-AA research framework: toward a biological definition of alzheimer’s disease. Alzheimers Dement.

[18] Ji C, Sigurdsson EM (2021). Current status of clinical trials on tau immunotherapies. Drugs.

[19] Jönsson T, Atwal JK, Steinberg S (2012). A mutation in APP protects against Alzheimer’s disease and age-related cognitive decline. Nature.

[20] McKhann GM (2011). The diagnosis of dementia due to Alzheimer’s disease: recommendations from the National Institute on Aging-Alzheimer’s Association workgroups on diagnostic guidelines for Alzheimer’s disease. Alzheimers Dement.

[21] Mintun MA, Lo AC, Duggan Evans C (2021). Donanemab in early Alzheimer’s disease. N Engl J Med.

[22] Novak P, Kovacech B, Katina S (2021). ADAMANT: a placebo-controlled randomized phase 2 study of AADvac1, an active immunotherapy against pathological tau in Alzheimer’s disease. Nat Aging.

[23] Ostrowitzki S, Lasser RA, Dorflinger E (2017). A phase III randomized trial of gantenerumab in prodromal Alzheimer’s disease. Alzheimers Res Ther.

[24] Polanco JC, Li C, Bodea LG (2018). Amyloid-β and tau complexity—towards improved biomarkers and targeted therapies. Nat Rev Neurol.

[25] Scheltens P, De Strooper B, Kivipelto M (2021). Alzheimer’s disease. Lancet.

[26] Schulman KA, Greicius MD, Richman B (2021). Will CMS find aducanumab reasonable and necessary for alzheimer disease after FDA approval?. JAMA.

[27] Schwarz S, Frölich L, Burns A (2012). Pharmacological treatment of dementia. Curr Opin Psychiatry.

[28] Selkoe DJ, Hardy J (2016). The amyloid hypothesis of Alzheimer’s disease at 25 years. EMBO Mol Med.

[29] Swanson CJ, Zhang Y, Dhadda S (2021). A randomized, double-blind, phase 2b proof-of-concept clinical trial in early Alzheimer’s disease with lecanemab, an anti-Aβ protofibril antibody. Alzheimers Res Ther.

[30] Vermunt L, Sikkes SAM, van den Hout A (2019). Duration of preclinical, prodromal, and dementia stages of Alzheimer’s disease in relation to age, sex, and APOE genotype. Alzheimers Dement.

[31] Vogelgsang J, Wiltfang J (2019). Neue Biomarker für die Alzheimer-Krankheit in Liquor und Blut [New biomarkers for Alzheimer’s disease in cerebrospinal fluid and blood]. Nervenarzt.

[32] Wang X, Sun G, Feng T (2019). Sodium oligomannate therapeutically remodels gut microbiota and suppresses gut bacterial amino acids-shaped neuroinflammation to inhibit Alzheimer’s disease progression. Cell Res.

[33] Wilcock GK, Gauthier S, Frisoni GB (2017). Potential of low dose leuco-methylthioninium Bis(Hydromethanesulphonate) (LMTM) monotherapy for treatment of mild alzheimer’s disease: cohort analysis as modified primary outcome in a phase III clinical trial. J. Alzheimers Dis..

