# Erratum zu: Krankheitsmodifizierende Therapieansätze bei Alzheimer-Krankheit

**DOI:** 10.1007/s00115-022-01366-3

**Published:** 2022-08-12

**Authors:** Lutz Frölich, Lucrezia Hausner

**Affiliations:** Zentralinstitut für Seelische Gesundheit Mannheim, Abteilung Gerontopsychiatrie, Universität Heidelberg, Quadrat J5, 68159 Mannheim, Deutschland

**Erratum zu:**

**Nervenarzt 2021**

10.1007/s00115-021-01222-w

Der Inhaber des Copyrights wurde fälschlicherweise mit „Springer Medizin Verlag GmbH, ein Teil von Springer Nature 2021“ angegeben. Richtig ist „Der/die Autor(en) 2021“.

Weiterhin fehlte in diesem Artikel die Lizenzangabe. Sie hätte „Open Access. Dieser Artikel wird unter der Creative Commons Namensnennung 4.0 International Lizenz veröffentlicht, welche die Nutzung, Vervielfältigung, Bearbeitung, Verbreitung und Wiedergabe in jeglichem Medium und Format erlaubt, sofern Sie den/die ursprünglichen Autor(en) und die Quelle ordnungsgemäß nennen, einen Link zur Creative Commons Lizenz beifügen und angeben, ob Änderungen vorgenommen wurden. Die in diesem Artikel enthaltenen Bilder und sonstiges Drittmaterial unterliegen ebenfalls der genannten Creative Commons Lizenz, sofern sich aus der Abbildungslegende nichts anderes ergibt. Sofern das betreffende Material nicht unter der genannten Creative Commons Lizenz steht und die betreffende Handlung nicht nach gesetzlichen Vorschriften erlaubt ist, ist für die oben aufgeführten Weiterverwendungen des Materials die Einwilligung des jeweiligen Rechteinhabers einzuholen. Weitere Details zur Lizenz entnehmen Sie bitte der Lizenzinformation auf http://creativecommons.org/licenses/by/4.0/deed.de.“ lauten sollen.

Der Originalbeitrag wurde korrigiert.

